# Multi-Drug Resistant Plasmids with ESBL/AmpC and *mcr-5.1* in Paraguayan Poultry Farms: The Linkage of Antibiotic Resistance and Hatcheries

**DOI:** 10.3390/microorganisms9040866

**Published:** 2021-04-17

**Authors:** Kristina Nesporova, Adam Valcek, Costas Papagiannitsis, Iva Kutilova, Ivana Jamborova, Lenka Davidova-Gerzova, Ibrahim Bitar, Jaroslav Hrabak, Ivan Literak, Monika Dolejska

**Affiliations:** 1 CEITEC VETUNI, University of Veterinary Sciences Brno, 61242 Brno, Czech Republic; krisnesporova@gmail.com (K.N.); valcekam@gmail.com (A.V.); kutilova.iva@gmail.com (I.K.); ivcajamborova@gmail.com (I.J.); ldavidova.gerzova@gmail.com (L.D.-G.); literaki@vfu.cz (I.L.); 2 Department of Biology and Wildlife Diseases, Faculty of Veterinary Hygiene and Ecology, University of Veterinary Sciences Brno, 61242 Brno, Czech Republic; 3 Biomedical Centre, Charles University, 32300 Pilsen, Czech Republic; ibrahimbitar5@gmail.com (I.B.); jaroslav.hrabak@lfp.cuni.cz (J.H.); 4 Department of Microbiology, University Hospital of Larissa, 41110 Larissa, Greece; c.papagiannitsis@gmail.com; 5 Faculty of Medicine in Pilsen, Charles University, 30605 Pilsen, Czech Republic

**Keywords:** ESBL/AmpC, colistin, *E. coli*, *K. pneumoniae*, I1 plasmids

## Abstract

Poultry represents a common source of bacteria with resistance to antibiotics including the critically important ones. Selective cultivation using colistin, cefotaxime and meropenem was performed for 66 chicken samples coming from 12 farms in Paraguay while two breeding companies supplied the farms. A total of 62 *Escherichia coli* and 22 *Klebsiella pneumoniae* isolates were obtained and representative isolates were subjected to whole-genome sequencing. Relatively high prevalence of phylogenetic group D and F was observed in *E. coli* isolates and several zoonotic sequence types (STs) including ST457 (14 isolates), ST38 (5), ST10 (2), ST117 (2) or ST93 (4) were detected. Isolates from three farms, which purchased chicken from a Paraguayan hatchery showed higher prevalence of *mcr-5.1* and *bla*_CTX-M-8_ compared to the other nine farms, which purchased chickens from a Brazilian hatchery. Moreover, none of the *K. pneumoniae* isolates were linked to the Paraguayan hatchery. ESBL/AmpC and *mcr-5*-carrying multi-drug resistant (MDR) plasmids were characterized, and complete sequences were obtained for eight plasmids. The study shed light on Paraguayan poultry farms as a reservoir of antibiotic resistance commonly conferred via MDR plasmids and showed linkage between resistance and origin of the chickens at the hatcheries level.

## 1. Introduction

Antibiotic resistance is growing continuously in various hosts and environments worldwide [1]. Bacteria resistant to critically important antibiotics are causing problems with treatment of severe infections in patients and represent only the tip of the iceberg as many different reservoirs of such bacteria including healthy food-producing animals have been identified [2,3]. Exchange routes of antibiotic resistant bacteria between animals and humans have not yet been resolved, with various studies suggesting possible scenarios [4,5]. Therefore, increasing incidence of multi-drug resistant (MDR) bacteria in the food production industry, especially of isolates belonging to the *Enterobacterales* family, is highly concerning [1,6]. Their transmission to the human population represents a threat for public health and may reduce the treatment options if they cause an infection.

Extended-spectrum beta-lactamases (ESBL) and AmpC-type beta-lactamases (AmpC) causing resistance to third generation cephalosporins (3GC) are often found in poultry production [6]. Moreover, the bacteria found in chickens are commonly MDR and carry resistance genes for several antibiotic groups [7]. 

Recently, several variants of plasmid-mediated *mcr* genes encoding resistance to colistin have been described [8]. The emergence of *mcr* genes compromises the use of colistin, which has been considered as a last resort antibiotic [8]. Isolates carrying *mcr-1* are often associated with ESBL/AmpC or carbapenemase-encoding genes. Such MDR isolates were detected in Brazil, Colombia, and Argentina [9,10], likely reflecting high prevalence of ESBL/AmpC-producing Gram-negative bacteria in South America. In these countries plasmid-mediated *bla*_CTX-M_ (e.g., *bla*_CTX-M-2, -8, -9, -15, -59_) and *bla*_CMY-2_ genes are predominating, and the poultry industry is considered as a reservoir for these genes [7,11] even in countries where 3GC administration is not authorized in poultry [6].

A previous study from our group reported the identification of 28 *mcr-5*-carrying isolates recovered from Paraguayan chicken farms which are included in this study [12]. Two more isolates of *E. coli* sequence type (ST) 457 were already presented in a study focused on this ST [13]. Nevertheless, these data are crucial to provide the overall picture of resistant bacteria detected in Paraguayan poultry among different farms and their relationships as no such study has been conducted before in Paraguay. Here, we provide a comparison of all, mostly MDR, isolates recovered from the chicken farms, and investigate linkage of detected antibiotic resistance genes (ARGs). Additional aim was to characterize plasmids responsible for the spread of ESBL/AmpC-encoding genes in Paraguayan poultry, provide deeper insights into the plasmid epidemiology and further examine *mcr-5.1*-carrying plasmids detected previously [12]. 

## 2. Materials and Methods

### 2.1. Samples Collection and Selective Cultivation

The experiment was performed according to the guidelines of the Ethic Committee of University of Veterinary Sciences Brno. Overall, 66 individual fecal samples were collected in 2012 from finisher broilers (28-days old) at 12 chicken farms in Paraguay in six different locations close to Asunción as described previously [12]. The farms purchased chicken from two different hatcheries/breeding companies (in Paraguay and in Brazil). No sampling was done directly at the hatcheries. All samples were cultivated on three plates of MacConkey agar (Oxoid, Basingstoke, UK) with cefotaxime (2 mg/L), colistin (3.5 mg/L) or meropenem (0.125 mg/L with addition of 100 mg/L zinc sulphate) to obtain isolates with emerging antibiotic resistance phenotypes. A single colony of *E. coli* or *Klebsiella* spp. morphology per plate was taken and cultivated to obtain pure cultures. All isolates were species-identified using MALDI-TOF MS (Bruker Daltonics, Bremen, Germany) and DNA from each was isolated using heat lysis. 

### 2.2. Antibiotic Susceptibility Testing 

Susceptibility to 12 antimicrobial substances was tested for all isolates using disk diffusion method on Mueller-Hinton agar (Oxoid, Basingstoke, UK) as previously described using Clinical and Laboratory Standards Institute (CLSI) guidelines [12]. 

### 2.3. Clonality of K. pneumoniae Isolates and Testing of Selected Resistance Genes

Clonal relationships among *K. pneumoniae* isolates were evaluated using XbaI Pulsed-field electrophoresis (PFGE) followed by the analysis of macrorestriction patterns using BioNumerics software v. 6.6 (Applied Maths, Ghent, Belgium). PFGE results were used to select representative isolates for whole-genome sequencing (WGS). 

Only 15 out of 22 *K. pneumoniae* isolates were subjected to WGS as the remaining seven isolates showed 100% similarity in respective PFGE clusters (Figure S2). While WGS can be more specific than PFGE, we have observed only minor differences (0–5 SNPs) among sequenced isolates from the same cluster. The seven isolates were tested for presence of resistance genes to 3GC (*bla*_CTX-M_, *bla*_TEM_, *bla*_SHV_, *bla*_OXA_, *bla*_CMY_) and colistin (*mcr-1*, *mcr-2*, *mcr-3*, *mcr-4*, *mcr-5*) using polymerase-chain reaction (PCR) [14,15,16] and the variant of the gene was determined with Sanger sequencing (Macrogen, Amsterdam, The Netherlands).

### 2.4. Whole-Genome Sequencing and Data Analysis

*E. coli* was the most prevalent organism found in our study and as such it provided an opportunity to compare individual farms and hatcheries. All *E. coli* isolates were characterized using short-read based WGS. Additionally, a single *E. coli* isolate 1517k was long-read sequenced using PacBio, as described previously [13]. Selection of the *K. pneumoniae* isolates for WGS was based mainly on their origin and clonality. At least one isolate from each farm per cluster was taken for WGS (Figure S2). 

WGS was performed for a total of 62 *E. coli* (GenBank BioProject accession no. PRJNA513237 and PRJNA630550) and 15 *K. pneumoniae* isolates (GenBank BioProject accession no. PRJNA698801). Genomic DNAs for WGS were isolated using NucleoSpin^®^ Tissue kit (Macherey-Nagel GmbH & Co, Dueren, Germany). The libraries were prepared using Nextera XT DNA Sample Preparation Kit and sequenced on MiSeq (Illumina, San Diego, CA, USA) platform using MiSeq kit V2 (Illumina, San Diego, CA, USA). Raw sequencing data were quality (Q ≥ 20) and adaptor trimmed using Trimmomatic software (v. 0.36) [17] and assembled with SPAdes software (v. 3.11.1) [18]. Fasta files were filtered for minimal coverage of 30 and evaluated for the presence of resistance genes using the ResFinder (v. 3.0) [19] and assigned to sequence types using MLST (v. 2.0) [20]. The fasta files were analysed by ABRicate employing PlasmidFinder (v. 2.0) [21]. Online tool pMLST (v. 2.0) [21] was employed to reveal presence of plasmid replicons and their plasmid ST. The phylogenetic group (PG) of *E. coli* isolates was determined by ClermonTyping [22]. 

### 2.5. Phylogenetic and Genomic Analysis

Phylogenetic analysis was performed using CSI Phylogeny (v. 1.4) [23] with *E. coli* 1517k (GenBank accession no. CP054388) as a reference for *E. coli* and *K. pneumoniae* 1466e (GenBank BioSample accession no. SAMN17763068) for *K. pneumoniae.* Phylogenetic analysis of five isolates of *E. coli* ST38 in comparison to publicly available sequences of ST38 recovered from EnteroBase (https://enterobase.warwick.ac.uk/, accessed on 4 June 2020) was performed based on Prokka open reading frames prediction [24] and multi-fasta alignment using Roary (v.3.7.0) [25]. The preliminary tree was generated using RAxML [26] and included 995 strains of *E. coli* ST38. A total of 32 EnteroBase strains were selected to perform a more specific phylogenetic analysis with focus on strains closely related to our five Paraguayan isolates. 

The phylogenetic trees were visualized using iTOL with mid-rooted function [27]. Comparison of *E. coli* ST457 genomes linked to different hatcheries was performed employing progressive Mauve [28]. 

### 2.6. Transferability of ESBL/AmpC Genes 

The filter-mating method was used to perform conjugation experiments to evaluate the transferability of plasmids carrying ESBL/AmpC genes in selected isolates which possess these genes according to PCR testing and WGS results. Plasmid-free laboratory strain *E. coli* MT102 resistant to sodium azide and rifampicin was used as a recipient. 

Transconjugants (Tc) were selected on Luria-Bertani agar (Oxoid, Basingstoke, UK) with sodium azide (100 mg/L), rifampicin (25 mg/L) and cefotaxime (2 mg/L). Successful transfer of the ESBL/AmpC genes to the recipient was confirmed by PCR testing. Transferability of *mcr* genes was evaluated, as described previously [12]. 

### 2.7. Typing of Plasmids Carrying ESBL/AmpC Genes

Representative transconjugants were tested using S1-PFGE to reveal the number of plasmids and their approximate sizes. Only transconjugants with single plasmids were included into the further testing. Results of PCR-based replicon typing (PBRT) [29] and pMLST online tool [21] of the donors were used to assign plasmids into incompatibility groups and plasmid sequence types, respectively. The presence of *mcr* and ESBL/AmpC genes in transconjugants was tested by PCR.

### 2.8. Plasmid Sequencing and Data Analysis

Different strategies were used in order to obtain the information about plasmid structures with focus on the ones carrying *mcr-5.1*. Plasmid DNA from eleven single-plasmid transconjugants was isolated using Genopure Plasmid Midi Kit (Roche, Prague, Czech Republic) and sequenced on the MiSeq (Illumina, San Diego, CA, USA) platform. The transconjugants came from different wild type strains and were selected based on the assignment to plasmid replicon ST (using WGS data of the wild strains) together with PBRT and S1-PFGE results to cover supposed *mcr-5*-carrying plasmid types. This strategy allowed us to obtain data about additional resistance genes carried on the same plasmid besides *mcr-5.1*. However, with exception of 1522kTc1, plasmid sequences were obtained in multiple contigs making the plasmid closing using these data challenging, as no reference plasmids similar to ours with *mcr-5.1* were found in public databases. 

Therefore, PacBio sequencing was employed for plasmid DNA from transconjugants 1514kTc1, 1524kTc1 and for genomic DNA from transconjugants 1512eTc1, 1520kTc1 and 1525eTc1. Moreover, we obtained two closed plasmids from PacBio and Illumina data of 1517k wild strain resolved using Unicycler [30]. A single-contig plasmid of 1522kTc1 obtained from Illumina was closed using PCR and Sanger sequencing (Macrogen, Amsterdam, The Netherlands). Plasmids of the same plasmid ST were aligned using BRIG [31]. 

## 3. Results and Discussion

### 3.1. Trends in Antibiotic Resistance among Paraguayan Poultry

**Figure 1:**
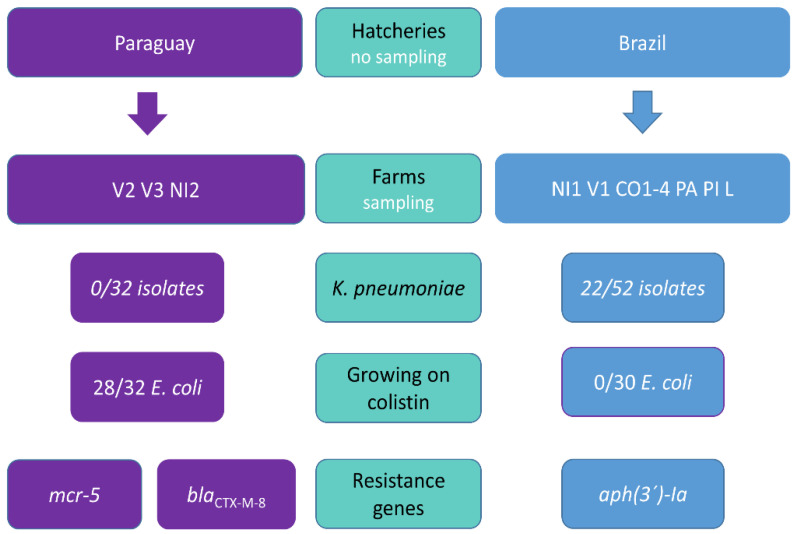
Schematic comparison of farms which purchased chickens from the Paraguayan hatchery and from the Brazilian hatchery regarding the most important findings suggesting that the hatchery level could play an important role in the chicken gut colonization and related antibiotic resistance carried by the bacteria. One-day chickens were transferred to the farms from hatcheries and sampled as finisher chickens (28-days old). Farm labels consist of the village name and farm number (Villeta: V1–3; Nueva Italia: NI1-2; Colonel Oviedo: CO1–4; Paraguari: PA; Piribebuy: PI; Luque: L). The scheme compares the number of obtained *Klebsiella pneumoniae* isolates to total number of obtained isolates in farms which purchased chicken from respective hatchery; the ability of *E. coli* isolates to grow on media with 3.5 mg/L colistin; and shows which antibiotic resistance genes presence clearly differed with respect to the hatchery, placing the specific gene with the respective hatchery for which it was typical.

This study is focused on the evaluation of resistance to critically important antibiotics including 3GC, colistin and carbapenems in Paraguayan chicken farms (Figure 1).

**Figure 2:**
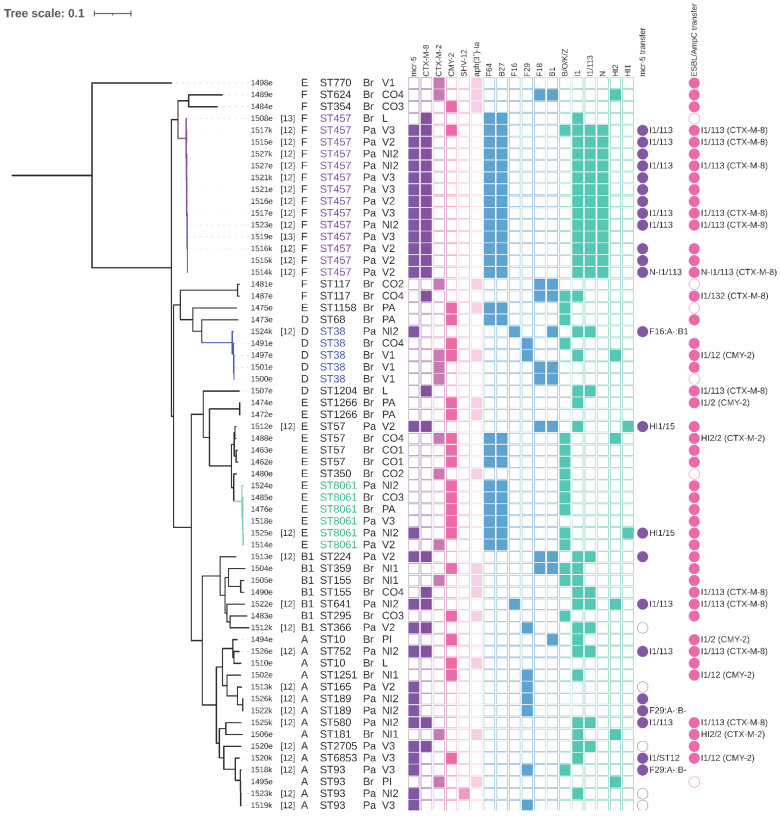
Phylogeny of *Escherichia coli* from Paraguayan farms. The most prevalent *E. coli* sequence types (STs) are highlighted in color. The columns represent isolate name (consist of the sample number and “k” for selection using colistin and “e” for selection using cefotaxime); reference number in case the isolate has been already part of previous publications; phylogenetic group; sequence type; hatchery (Br: Brazil, Pa: Paraguay) and farm (label consist of the village name and farm number: Villeta: V1–3; Nueva Italia: NI1-2; Colonel Oviedo: CO1–4; Paraguari: PA; Piribebuy: PI; Luque: L). The coloured squares indicate presence of selected antibiotic resistance genes (purple-pink), F plasmid replicons (blue) and other plasmids (turquoise). The colour circles indicate transferability of *mcr-5.1* (purple) or extended-spectrum beta-lactamases (ESBL) and AmpC-type beta-lactamases (AmpC) encoding genes (pink) in conjugation experiments, a full circle means the conjugation was successful including confirmation of presence of respective genes by polymerase –chain reaction (PCR), empty circles represent unsuccessful conjugation experiments. For the single-plasmid fully-typed transconjugants, the information about which plasmid replicon carried the respective gene is presented next to the circle.

**Figure 3:**
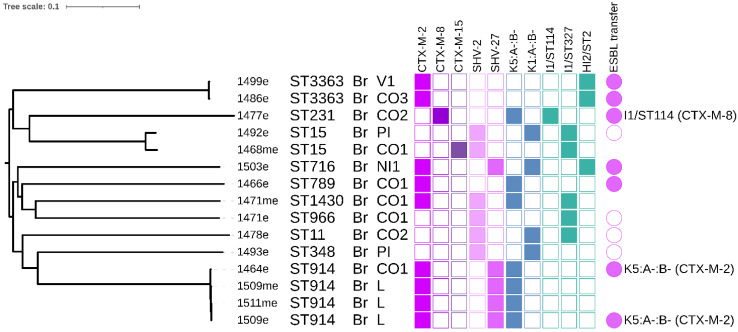
Phylogeny of selected *K. pneumoniae* isolates from Paraguayan farms. The columns isolate name (consist of the sample number and “me” for selection using meropenem and “e” for selection using cefotaxime); sequence type, hatchery (Br–Brazil); farm (label consist of the village name and farm number: Villeta: V1; Nueva Italia: NI1; Colonel Oviedo CO1–3; Piribebuy: PI; Luque: L). The color squares indicate presence of selected antibiotic resistance genes (purple-pink), F plasmid replicons (blue) and other plasmids (turquoise). The color circles indicate transferability of ESBL genes (pink) in conjugation experiments, full circles mean the conjugation was successful including confirmation of presence of respective genes by PCR, empty circles represent unsuccessful conjugation. For the single-plasmid fully typed transconjugants, the information which plasmid replicon carries the respective gene is presented next to the circle.

We obtained 62 *E. coli* (Figure 2) and 22 *K. pneumoniae* isolates. Sixteen of the *E. coli* isolates originated from media with colistin, while the rest were obtained on media with cefotaxime. *K. pneumoniae* isolates were obtained from media with cefotaxime (18 isolates) or meropenem (four isolates) but carbapenemase encoding genes were not detected in any of them (Figure 3). *K. pneumoniae* isolates came only from the farms which purchased chickens from the Brazilian breeding company (Figure 1 and Figure 3).

Most of the isolates (96%; 81/84) showed phenotypic resistance to three or more antibiotic groups which included beta-lactams, aminoglycosides, sulphonamides, tetracyclines, quinolones, trimethoprim and amphenicols, and were classified as MDR (Figures S1 and S2). Resistance to colistin was observed in 29 (out of 62) of *E. coli* isolates and WGS revealed these colistin-resistant isolates carried the *mcr-5.1* gene [12]. None of the *K. pneumoniae* isolates carried *mcr-5.1*. A total of 78 (out of 84) isolates resistant to 3GS were obtained from different media from 65 (98%; 65/66) chicken samples. The most prevalent gene variants encoding for resistance to 3GC detected in 62 *E. coli* isolates were *bla*_CTX-M-8_ (24/62), *bla*_CMY-2_ (22/62) and *bla*_CTX-M-2_ (12/62) (Figure 2). Overall, six *E. coli* isolates carried none of the ESBL/AmpC genes and two isolates carried two genes, *bla*_CMY-2_ and *bla*_CTX-M-2_ (Figure 2). In 22 *K. pneumoniae* isolates, *bla*_CTX-M-2_ (14/22), *bla*_SHV-27_ (9/22) and *bla*_SHV-2_ (5/22) were present most often and 11 of these isolates carried two ESBL genes (Figure 3).

Different farms from the same village (Villeta and Nueva Italia) with chickens from different hatcheries did not seem to show the same patterns in ARGs (Figure 1). The prevalence of some ARGs clearly varied among *E. coli* isolates recovered from chicken purchased from different breeding companies. Specifically, the difference was evident for *mcr-5.1*, *bla*_CTX-M-8_, and *aph(3’)-Ia*, while the first two genes were typically detected in isolates from the Paraguayan breed and the last gene was specific for the Brazilian breed. This suggests that hatcheries, particularly on the first day of chicken life, may play a crucial role in the development of the chicken microbiome and that the early-stage obtained bacteria and ARGs patterns may tend to be maintained. While the study was not designed to deal with this question and several key pieces of information are missing (including usage of antibiotics at hatcheries and farms), the distribution of observed colistin resistance which was linked only to the Paraguayan breed together with lack of *K. pneumoniae* isolates in farms which took chickens from Paraguay and clonal dissemination of *E. coli* ST457 within all *mcr*-related farms support this assumption (Figure 1 and Figure 2). It was previously observed in chickens in Ecuador that young poultry which arrive at farms are already colonized with antibiotic resistant bacteria and the prevalence of the resistance tends to decline in older chickens, which suggests that the resistance was not acquired in the village or as a result of specific farms practice [32]. Similar results were already reported in France where the proportion of isolates from 283 farms (pooled samples) which were non-susceptible to 3GC differed significantly in relation to origin from six different hatcheries [33]. However, a recent review by Dame-Korevaar et al. [34], which aimed to present an overview of possible transmission routes of ESBL/AmpC-producing bacteria in the broiler production pyramid described that multiple transmission routes have been reported in included studies [34]. Therefore, the role of chicken hatcheries in antibiotic resistance transmission should be examined further as other factors may affect the hatcheries influence. 

### 3.2. Phylogenetic Relationships among E. coli Isolates

Regarding the clonal relationships of *E. coli* isolates, 29 different sequence types and diverse phylogenetic groups were detected among 62 isolates including F (29%, 18/62), A (24%, 15/62), E (24%, 15/62), B1 (11%, 7/62) and D (11% 7/62). Strains from PG D are more likely to cause infections in humans and poultry strains from PG F were recently associated with human extraintestinal pathogenic *E. coli* (ExPEC) pathotypes [35,36]. Thus, high prevalence of strains from these PG among Paraguayan poultry is concerning. Moreover, several STs known for their capability to cause human infection were detected including ST457 (14 isolates), ST10 (n = 2), ST38 (n = 5), ST117 (n = 2) and ST93 (n = 4) [37,38]. Single *E. coli* isolates belonging to STs 1158 and 1251, which are considered to be international zoonotic sequence types, were present [39]. 

The most prevalent ST was *E. coli* ST457 of phylogenetic group F (23%, 14/62). Other prevalent *E. coli* STs included ST8061 (10%, 6/62), which was firstly detected previously within our Paraguayan samples [12] and ST38 (8%, 5/62). 

### 3.3. E. coli ST457 from Paraguayan and Brazilian Hatcheries 

*E. coli* ST457 linked to the Paraguayan hatchery is an example of a clonal spread of a successful lineage as it represents 41% (13/32) of all strains linked to this hatchery and it was found in each of the three farms purchasing chickens from this hatchery. On the other hand, a single isolate of ST457 was detected in a chicken linked to the Brazilian hatchery. This isolate carried the same beta-lactamase gene (*bla*_CTX-M-8_) but it lacked *mcr-5.1*. It is possible that the *mcr-5.1* gene acquisition in the conditions of the Paraguayan breed could be beneficial for spread of the ST457 clone, however genomic comparison of 1517k (PacBio-sequenced ST457 from the Paraguayan breed) and 1508e (ST457 from the Brazilian breed) strains showed differences also at the chromosomal level (Table S1). The main difference represents an approx. 33-kb region carried by Paraguayan strains with multi-drug efflux system and various metabolism-related genes predicted by RASTtk (Table S1, region D). 

We have already compared our Paraguayan *E. coli* ST457 isolates to its global cohort suggesting ST457 is a globally disseminated zoonotic lineage [13]. Moreover, a recent study focused on antibiotic resistance in Italian poultry detected ST457 as the most prevalent ST, despite carrying different ARGs than our Paraguayan collection [6]. A recent study on diseased pigs in the USA described ST457 as one of the most prevalent ST with closely related isolates found in diverse sources including humans [40]. 

### 3.4. E. coli ST38 Comparison

We focused also on ST38 which is a common *E. coli* lineage capable of causing ExPEC disease and it was the third most common ST in our collection [37]. While the spread of *E. coli* ST38 carrying *bla*_OXA-48_ is particularly worrying [41], ST38 is also an important vector for spread of ESBL genes as it was detected in diverse sources including wild birds from Mongolia and Brazil [39,42].

The phylogenetic analysis of our ST38 strains and those retrieved from EnteroBase revealed that strain 1524k associated with the Paraguayan hatchery is less related to four strains associated with the Brazilian hatchery (275–277 SNPs difference) than some other strains detected globally while all four Brazilian strains are clonal (1–5 SNPs) despite coming from two different farms and villages (Figure S3, Figure 2). Interestingly, the Brazilian strains were very closely related to the strain SCP05-44 (ESC_EA9861AA) of human origin from the Netherlands (24–25 SNPs) recovered from EnteroBase (Figure S3). The Dutch strain even showed similarities in resistance and plasmid profiles to the Brazilian strains (Figure S3). This finding highlights that very closely related bacteria may occupy diverse niches including human without being strictly source-accommodated by carriage of host-specific genes. 

### 3.5. K. pneumoniae Phylogeny 

The most prevalent *K. pneumoniae* was ST914 as 8 out of 22 isolates belonged to the clonal cluster. This ST was detected within two different farms which purchased chicken from the Brazilian breed. The second most prevalent *K. pneumoniae* ST was ST3363 (3 isolates). Four isolates of ST914 were sequenced using Illumina and differed in 0–3 SNPs (Figure 3, Figure S2). All of them carried *bla*_CTX-M-2_ which was located on a conjugative F plasmid with K5:A-:B- replicon. *K. pneumoniae* ST914 has been previously detected in a study focusing on biofilm formation from a hospital in China [43] and in a Bulgarian chicken [44]. 

Two *K. pneumoniae* isolates belonging to ST15 were detected in our chicken collection, notably one of them carried *bla*_CTX-M-15_. *K. pneumoniae* ST15 producing CTX-M-15 has spread worldwide and is considered to be a clinically associated lineage [45]. It has been detected in many sources including human patients [46], companion animals [45] and poultry [44]. 

### 3.6. Conjugation Experiments and Plasmid Typing

The conjugation experiments revealed that ESBL/AmpC genes were transferred successfully from 88% (46/52) selected isolates of *E. coli* (Figure 2). As was described previously, *mcr-5.1* was successfully transferred in 82% (23/28) of selected *E. coli* isolates [12]. Conjugation transfer of ESBL genes was successful in 75% (12/16) of selected *K. pneumoniae* isolates (Figure 3 and Figure S2). 

Further typing of single-plasmid transconjugants revealed that several replicons are involved in carriage of ARGs of interest and that some I1 plasmids carried combination of *mcr-5.1* and ESBL/AmpC genes (Figure 2). We detected I1/ST113-*bla*_CTX-M-8_-*mcr-5*; I1/ST12-*bla*_CMY-2_-*mcr-5*; a fusion N-I1/ST113-*bla*_CTX-M-8_-*mcr-5*; F16:A:-B1-*mcr-5*; F29:A:-B-*mcr-5*; HI1/ST15-*mcr-5* from *E. coli* coming from the Paraguayan breed (Figure 2). Interestingly, I1 plasmids of ST113 and ST12 were present in *E. coli* linked to the Brazilian hatchery as well but they carried only respective ESBL/AmpC genes: I1/ST113-*bla*_CTX-M-8_ and I1/ST12-*bla*_CMY-2_ (Figure 2). Other conjugative plasmid replicons from *E. coli* of the Brazilian breed were I1/ST114-*bla*_CTX-M-8_, I1/ST132-*bla*_CTX-M-8_, I1/ST2-*bla*_CMY-2_ and HI2/ST2-*bla*_CTX-M-2_ (Figure 2). 

The plasmid profiles of *K. pneumoniae* isolates typically differ from *E. coli* isolates in our set however, *K. pneumoniae* isolate 1477e carried I1/ST114-*bla*_CTX-M-8_ which suggests the same plasmid type may be shared interspecies to some extent. Besides that, we detected one F plasmid of K5:A-:B-*bla*_CTX-M-2_ in *K. pneumoniae* isolates (Figure 3).

### 3.7. Detected Plasmid Replicons and mcr-5-Carrying Plasmids

In total, we detected more than 30 different plasmid replicons in *E. coli* isolates using the PlasmidFinder tool (Figure S1). We have previously reported occurrence of *mcr-5.1* in various conjugative plasmid types and suggested the whole transposon with *mcr-5.1* is highly transferable [12]. To expand our knowledge about the possible vectors of relatively newly identified *mcr*-*5.1*, we have focused mainly on the structure of plasmids carrying this gene and its association with other resistance genes, particularly ESBL/AmpC.

We have obtained completely assembled eight (six with *mcr-5.1*) plasmids in total (Table 1) which were used as a reference for the comparison of related plasmids detected in our collection or related plasmids from other studies using BLASTn. 

### 3.8. I1/ST113 and I1/ST12 Plasmids

IncI1/ST113 stood out with its high prevalence (35%, 22/62) in *E. coli*. All I1/ST113 carried *bla*_CTX-M-8_ and most of them carried *mcr-5.1* as well. 

I1/ST1113 plasmids were present in all thirteen *E. coli* ST457 isolates coming from the Paraguayan breed. The isolates carried the N replicon with undetermined plasmid ST (*repN-1*, *traJ-8*, *korA* not present) as well. Interestingly, plasmid from 1514k (ST457) was a fusion comprised of N-I1/ST113 replicons but the major population of other ST457 isolates carried I1/ST113 and N plasmid separately as was revealed using sequencing of plasmid DNA from transconjugants, S1-PFGE and PBRT typing of transconjugants. 

**Figure 4:**
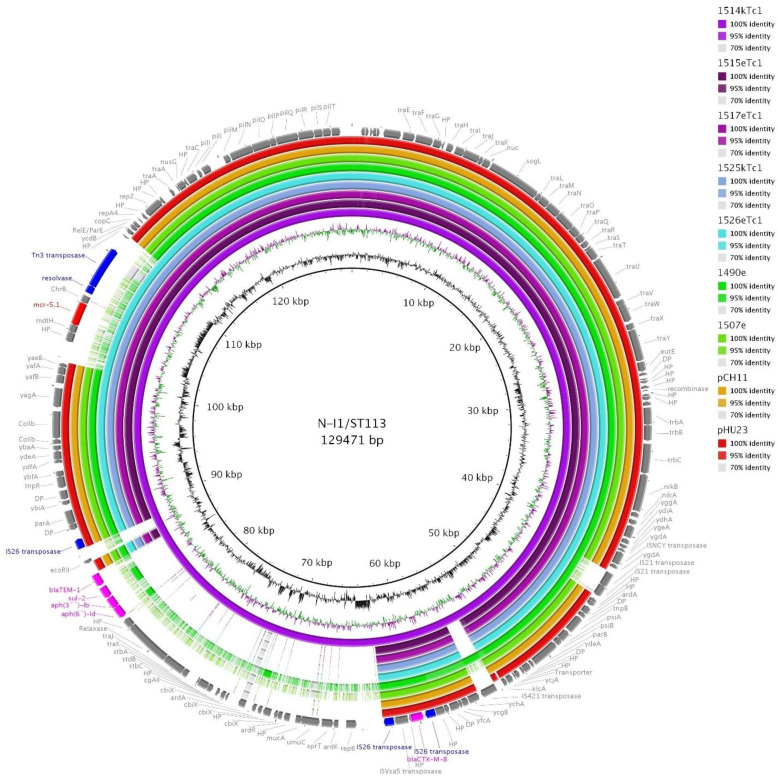
BRIG comparison of selected I1/ST113 plasmids. Depicted I1/ST113 plasmids came from the Paraguayan hatchery isolates (1514kTc1, 1515eTc1,1517eTc1, 1525kTc1 and 1525eTc1 using Illumina data from plasmid-DNA of a single-plasmid transconjugant Tc1), from the Brazilian hatchery (1490e and 1507e) and a previous study from Japan (pCH11 and pHU23) [5]. A reference fusion N-I1/ST113 plasmid was obtained using PacBio (GenBank accession no MW800641). Antibiotic resistance genes are depicted in fuchsia, except *mcr-5.1* (red), mobile genetic elements of interest are highlighted in blue.

We used the fusion N-I1/ST113 plasmid from 1514k from PacBio sequencing as a reference (GenBank accession no. MW800641) for BRIG comparison (Figure 4). We included sequencing data obtained using plasmid DNA isolated from respective transconjugants of selected isolates—1514k, 1515e, 1517e (all *E. coli* ST457), 15125k (ST580), 1526e (ST752), WGS data from 1490e and 1507e isolates coming from the Brazilian breed (without *mcr-5*), and previously described I1/ST113-*bla*_CTX-M-8_ plasmids from human (pHU23) and from chicken (pCH11) of Japanese origin [5]. We selected only these plasmids because using WGS data from *E. coli* ST457 lead to a false depiction of all of I1/ST113 plasmids as fusion ones because they all carry both replicons I1/ST113 and N as illustrated in supplementary Figure S4. WGS data from isolates with other *E. coli* STs (ST38, ST57, ST224 and ST641) from the Paraguayan breed which carried *mcr-5.1* but not within I1/ST113 would be depicted as the part with *mcr-5.1* is present despite belonging to another background. All 22 isolates carrying I1/ST113 are compared using BRIG in supplementary Figure S4 to illustrate the issues, which may occur with using WGS Illumina data for plasmid alignment.

Two plasmids from the Japanese study were included in Figure 4 because the study suggested their occurrence in Japan might be related to import of chicken from South America [5]. The BRIG analysis showed the Paraguayan IncI1/ST113 plasmids contained identical regions with the Japanese IncI1/ST113 plasmids including plasmid backbone and the region with *bla*_CTX-M-8_ flanked by two copies of IS*26*. They also shared genes encoding for a nickel transport system, colicin-Ib immunity protein, ethanolamine utilization protein EutE, and the RelE/ParE family toxin-antitoxin system. The region with *mcr-5.1* of 7262 bp was previously described and compared with other plasmid types from Paraguay [12]. 

While *bla*_CTX-M-8_ on I1/ST113 plasmids has been commonly detected in Brazil in humans and animals so these plasmids are considered epidemic for this region [47,48], they were also found in Spain and Germany [49,50] and Japan [5]. Interestingly, the Japanese plasmids seemed to show very similar sequence to ours, especially with the plasmid from 1507e (*E. coli* ST1204) related to the Brazilian hatchery (Figure 4). Therefore, the epidemiological linkage between Japan and South America through the chicken import suggested by Norizuki et al. [5] may be relevant. 

**Figure 5:**
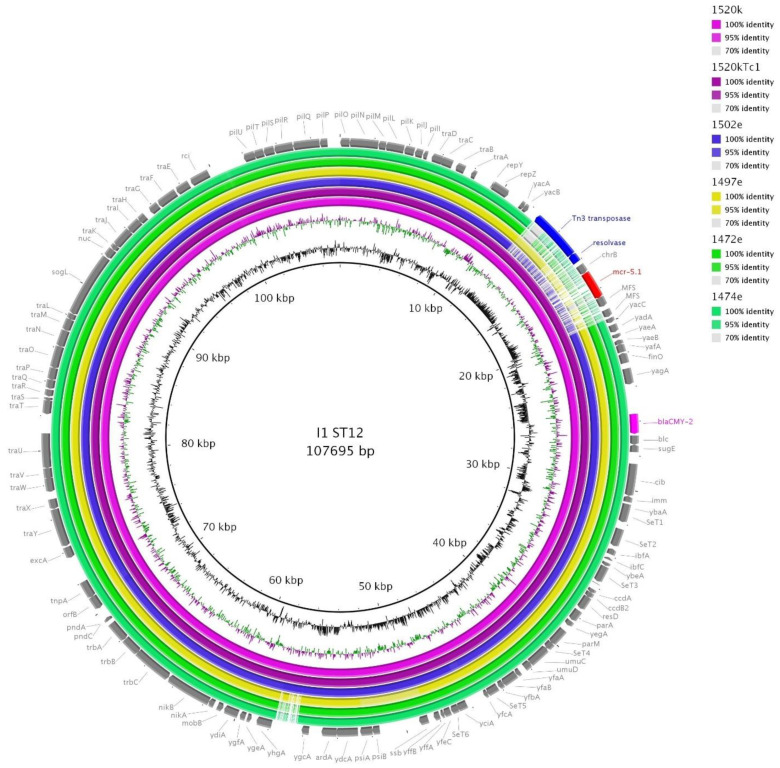
BRIG comparison of I1/ST12 plasmids. Reference plasmid 1520k (GenBank accession no. MW800639) obtained from PacBio was compared to Illumina data from wild type (1520k) and the respective transconjugant 1520kTc1), both purple circles. The comparison of five isolates with I1/ST12 reveals all I1/ST12 plasmids are quite conserved and share *bla*_CMY-2_ (fuchsia). They differed only in part with the transposon carrying *mcr-5.1* (red) in 1520k, mobile genetic elements of this transposon are highlighted in blue.

I1/ST12 plasmids carrying *bla*_CMY-2_ are found commonly in various sources including poultry and they are considered an epidemic plasmid lineage [51]. However, their combination with *mcr-5.1* has not been detected besides our strain 1520k of *E. coli* ST6853 and a respective reference plasmid I1/ST12-*bla*_CMY-2_-*mcr-5* (GenBank accession no. MW800639) [12]. Comparison of the reference plasmid with the other I1/ST12 plasmids linked to the Brazilian hatchery in isolates of *E. coli* ST1266, ST38 and ST1251 showed all I1/ST12-*bla*_CMY-2_ plasmids were very conserved and almost identical, with exception of the *mcr-5.1* region (Figure 5). However, WGS data were used for the comparison, therefore there could be some unrevealed differences. Considering the global success of I1/ST12-*bla*_CMY-2_, the incorporation of *mcr-5.1* resulting in co-resistance to 3GC and colistin carried by such a plasmid lineage is worrisome.

### 3.9. F64:A-:B27, F29:A-:B- and F16:A-:B1 Plasmids

Interestingly, the most prevalent replicon combination of F plasmids in *E. coli*, F64:A-:B:27 (40%, 25/62) did not carry any ARGs in our closed reference F64:A-:B:27 plasmid (GenBank accession no. MW800638) from *E. coli* ST457 (1517k), however it contained genes for efflux transport systems (MFS and RND type), colicin-production related genes (*cba*, *cia*, *cma*), virulence-associated genes such as *papABCDH* (fimbriae-related), *ompT* (outer membrane protease), *caf1M, caf1A* (F1 capsule-related), *afaD* (invasin), *ccdB* (toxin)*, hlyF* (hemolysin F), *traT* (outer membrane protein complement resistance) and metabolism and substrate uptake related genes (Figure S5). 

**Figure 6:**
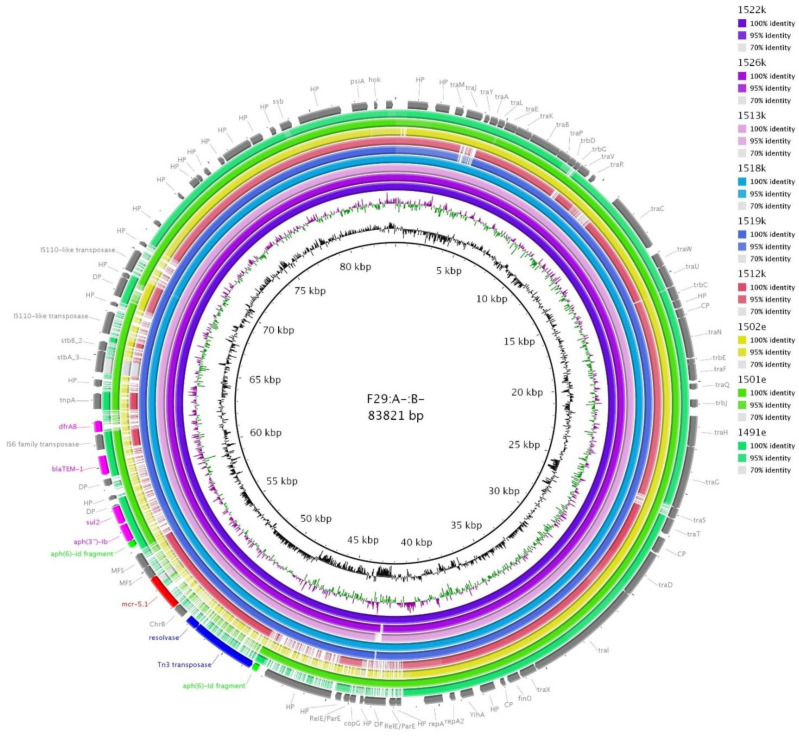
BRIG comparison of F29:A-:B- plasmids. Reference plasmid of F29:A-:B- was obtained from the 1522k strain (GenBank accession no. MW800637). Plasmids from the Paraguayan hatchery are depicted in purple, blue and red circles, while plasmids from the Brazilian hatchery are presented by the yellow and green circles. The transconjugants from the 1526k wild type strain was not evaluated using pulsed-field gel electrophoresis with S1 enzyme and PCR-based replicon typing because F replicon and *mcr-5.1* gene was present within the same contig in WGS data, therefore *mcr-5.1* was expected to be carried by F29:A-:B-. Antibiotic resistance genes are depicted in fuchsia, except *mcr-5.1* (red), and fragmented *aph(6)-Id* gene (lime) which was considered to be non-functional. Mobile genetic elements of the transposon with *mcr-5.1* are highlighted in blue.

Isolates 1522k (*E. coli* ST189) and 1518k (*E. coli* ST93) were identified as carrying *mcr-5.1* on F29:A-:B- plasmids together with *aph(3´´)-Ib, bla*_TEM-1_*, sul2* and *dfrA8* using Illumina data from single-plasmid transconjugants plasmid DNA. The transposon with *mcr-5.1* was incorporated into the plasmid in the way it truncated the *aph(6)-Id* gene (Figure 6). This suggest the acquisition of *mcr-5.1* happened later when at least some resistance genes were already present in F29:A-:B-. A closed sequence of F29:A-:B- plasmid (GenBank accession no. MW800637) was obtained for 1522k and used as a reference for BRIG comparison (Figure 6). Interestingly, the comparison in Figure 6 revealed two isolates, 1519k (*E. coli* ST93) and 1513k (*E. coli* ST165), with unsuccessful conjugation transfer of *mcr-5.1* contained un-interrupted region with *mcr-5.1* in their F29:A-:B- plasmids as well as one more strain (1526k, *E. coli* ST189) (Figure 6). The region with *mcr-5.1* was present as well in 1512k (using WGS data of wild strain for BRIG comparison), however, in this case, the surrounding of transposon with *mcr-5.1* was evidently truncated compared to the others. Therefore, *mcr-5.1* is probably not carried by F29:A-:B- plasmid in 1512k strain as the *mcr-5.1* region could be mapped from a different background. This suggests that besides an appreciable number of plasmid types which were identified as carrying *mcr-5.1* in Paraguayan chickens, other hitherto unknown vectors for *mcr-5.1* spread may be present.

We found two plasmids with a F16 replicon in *E. coli* ST38 isolate 1524k (F16:A-:B1) and *E. coli* ST155 isolate 1505e (F16:A-:B-), however their alignment revealed high variability except for the conserved plasmid backbone (Figure S6). A reference F16:A-:B1 plasmid (GenBank accession no. MW800635) from the 1524k strain was 142 664 bp long and carried six antibiotic resistance genes. The F16:A-:B1 plasmid found in *E. coli* pCys-6 (GenBank accession no. CP041301) shared 95% coverage and 99.98% identity with our reference plasmid using BLASTn. Plasmid pCys-6 came from urine of a patient with cystitis in Canada. MW800635 and pCys-6 plasmids shared a multi-resistance region of 29533 bp (99.98% identity) flanked by Tn*3*-family transposase genes and containing integrase gene *intI1* along with a mercury resistance operon and five ARGs including *dfrA12*, *aadA2*, *sul1*, *mph*(A) and *tet*(A) (Figure S6).

### 3.10. HI1 ST15 Plasmids

HI1 plasmids can be found in humans and animals with propensity to be MDR, therefore their spread represents a risk for public health [52]. We detected only two of those in our whole collection in *E. coli* ST57 isolate 1512e and *E. coli* ST8061 isolate 1525e. Both of the HI1 plasmids were assigned to a novel ST15. Their structure was quite unique as no similar plasmid was found using BLASTn. Both of them were MDR, carrying *mcr-5.1* and other ARGs encoding resistance to lincosamides [*lnu*(G)], tetracyclines [*tet*(B)] and sulphonamides (*sul2*), and a mercury resistance operon (Figure S7 and Table 1). A reference HI1 plasmid (GenBank accession no. MW800636) from 1525e possessed additional resistance genes *aph(6)-Id*, *aph(3´´)-Ib* (resistance to aminoglycosides) and *bla*_TEM-1_ (resistance to beta-lactams) compared to the other HI1 plasmid (Figure S7). 

## 4. Conclusions

Without a doubt, plasmids are very important features supporting bacterial survival under stress conditions, like the presence of antibiotics. This study reports novel information about the nucleotide sequences of conjugative *mcr-5*-carrying plasmids which were highly prevalent in *E. coli* from three different farms linked to the Paraguayan hatchery. These plasmids were compared to the plasmids of the same plasmid ST without *mcr-5* which were typically present in *E. coli* from the Brazilian hatchery (nine farms). Especially concerning is the description of colistin resistance in association with resistance to 3GC in epidemic plasmids I1/ST113 and I1/ST12 which commonly carry *bla*_CTX-M-8_ and *bla*_CMY-2_, respectively [5,51]. The I1/ST113 plasmids deserve more attention in future studies because of their zoonotic potential and possible spread via poultry/meat export. 

Similarly, as worrying as the high prevalence of resistance to 3GC and other antibiotic groups in Paraguayan chicken isolates is the relatively high prevalence of *E. coli* of phylogenetic group F and D together with detection of several globally emerging sequence types. *E. coli* ST457 has already showed its zoonotic and pathogenic potential and its capacity to outcompete the other *E. coli* STs in various, mostly avian, animal groups but also to cause infections in humans [13]. Our larger phylogenetic comparison of *E. coli* ST38 highlights the importance of a One Health attitude in dealing with the resistance issue as four of our strains were very closely related to the human isolate (ECS_EA9861AA) from the Netherlands. The notion that MDR bacteria know no boundaries, needs to be stressed. 

Above all, despite the study limitations including the low number of samples, our results suggest that hatcheries may have an important impact in determining antibiotic resistance carriage and in the selection of bacteria colonizing one day chickens in some cases. The influence of the different environment in their later life on the gut microbiota and resistance may be limited. More effort is needed to clearly identify the critical point of transmission of resistant bacteria in the poultry industry which may lead to finding how to influence it effectively to reduce the resistance in one of the major human food sources. Besides the antibiotic resistance itself, knowledge in the field of how to create and keep the “good” microbiome in chickens would be widely beneficial and have a positive economic impact, as the original problem was to avoid infections and deaths of chickens which secondarily created the antibiotic resistance issue.

## Figures and Tables

**Table 1:** Overview of plasmids with complete structure obtained in this study. The Approach column is specifying how the sequence was obtained, either by PacBio whole-genome sequencing (WGS) and combining the data with Illumina sequences using Unicycler (PacBio-WGS), by PacBio sequencing of respective single-plasmid transconjugant (PacBio-Tc), by PacBio sequencing of plasmid DNA extracted from a single-plasmid transconjugant (PacBio-pDNA) or using Illumina sequencing of Plasmid DNA extracted from single-plasmid transconjugants and closing using PCR (Illumina + PCR).

Isolate	Approach	Plasmid	Size (kbp)	Antibiotic Resistance Genes
1514k	PacBio-pDNA	N-I1/ST113	129.471	*mcr-5*, *bla*_CTX-M-8_, *bla*_TEM-1_, *aph(3´´)-Ib*, *aph(6)-Id*, *sul2*
1524k	PacBio-pDNA	F16:A-:B1	142.664	*mcr-5*, *aadA2*, *sul1*, *tet*(A), *dfrA12*
1520k	PacBio-Tc	I1/ST12	107.695	*mcr-5*, *bla*_CMY-2_
1512e	PacBio-Tc	HI1/ST15	197.568	*mcr-5*, *lnu*(G), *tet*(B), *sul2*
1525e	PacBio-Tc	HI1/ST15	209.411	*mcr-5*, *bla*_TEM-1_, *aph(3´´)-Ib*, *aph(6)-Id*, *lnu*(G), *tet*(B), *sul2*
1522k	Illumina + PCR	F29:A-:B-	83.821	*mcr-5*, *aph(3´´)-Ib*, *bla*_TEM-1_, *sul2*, *dfrA8*
1517k	PacBio-WGS	F64:A-:B27	167.074	-
1517k	PacBio-WGS	K2	86.723	*bla*_CMY-2_

## Data Availability

The data presented in this study are openly deposited in GenBank within BioProject PRJNA513237, PRJNA630550 and PRJNA698801 and the closed plasmid sequences are available in GenBank under accession numbers MW800634 (HI1/ST15 from 1512e), MW800635 (F16:A-:B1 from 1524k), MW800636 (HI1/ST15 from 1525e), MW800637 (F29:A-:B- from 1522k), MW800638 (F64:A-:B27 from 1517k), MW800639 (I1/ST12 from 1520k), MW800640 (K2 from 1517k) and MW800641 (N-I1/ST113 from 1514k).

## Supplementary Materials

The following are available online at https://www.mdpi.com/article/10.3390/microorganisms9040866/s1. The supplementary material is enclosed within a single file and contains Figure S1 (Phylogeny of *E. coli* isolates recovered in this study and all detected antibiotic resistance genes and plasmid replicons), Figure S2 (Phylogeny of selected *K. pneumoniae* isolates and table with data of non-sequenced *K. pneumoniae* isolates), Figure S3 (Phylogeny of *E. coli* ST38 – comparison of our isolates with closely related ones recovered from EnteroBase), Figure S4 (Comparison of I1/ST113 plasmids), Figure S5 (BRIG comparison of F64:A-:B27 plasmids), Figure S6 (BRIG comparison of F16 replicon plasmids in this study with closely related F16 plasmid recovered from GenBank), Figure S7 (BRIG comparison of HI1/ST15 plasmids), Table S1 (Comparison of 1517k genome (PacBio) and 1508e in progressiveMauve).
