# Supplementary material for: Multi-Drug Resistant Plasmids with ESBL/AmpC and mcr-5.1 in Paraguayan Poultry Farms: The Linkage of Antibiotic Resistance and Hatcheries
Source: Microorganisms. 2021 Apr 17;9(4):866. doi: 10.3390/microorganisms9040866 (PMC8072826; doi:10.3390/microorganisms9040866)
Supplement: Supplementary file 1 [file microorganisms-09-00866-s001.zip › microorganisms-1180204-supplementary.pdf]

Supplementary data

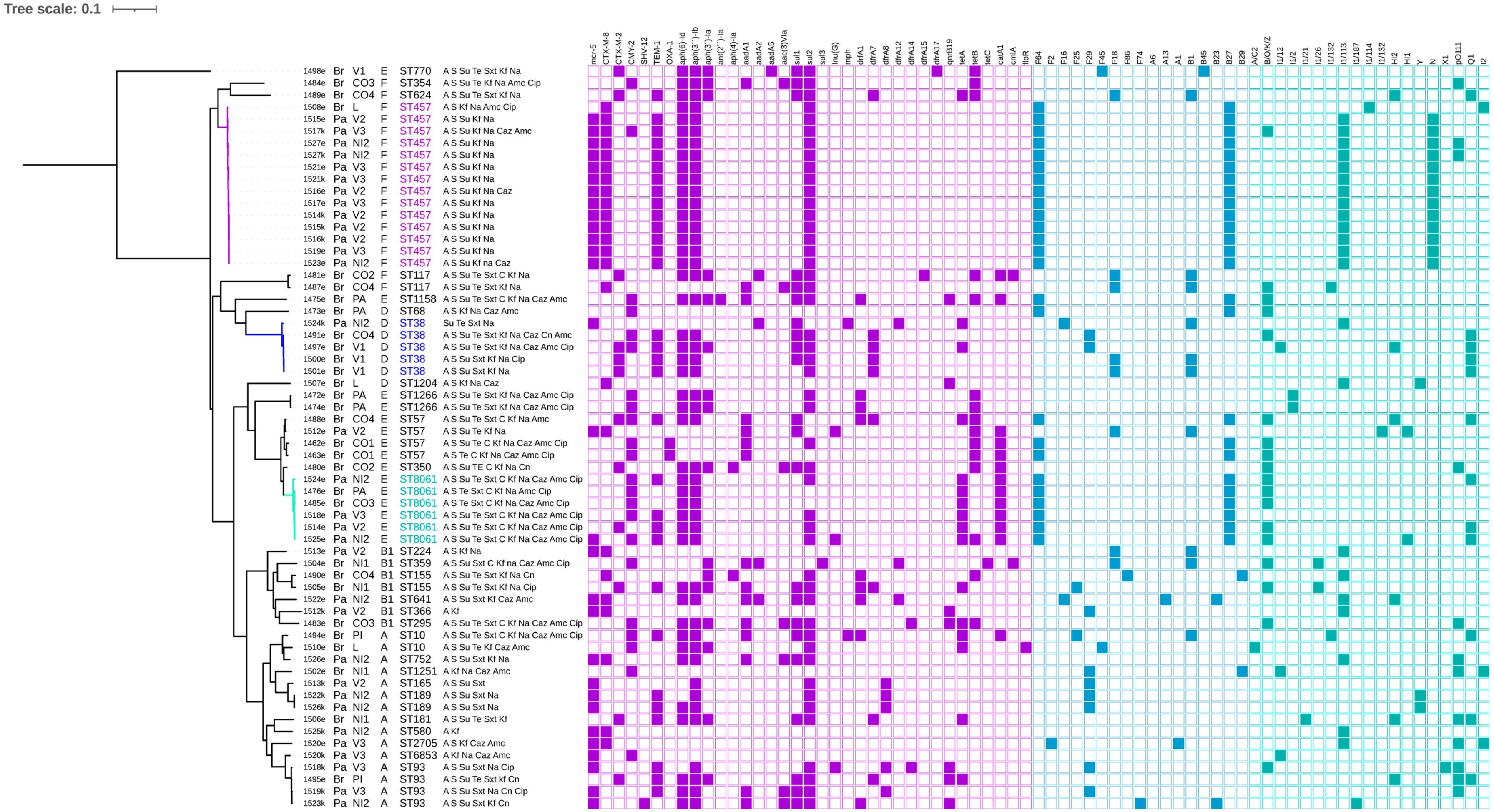

Figure S1. Phylogeny of *Escherichia coli* isolates recovered in this study and all detected antibiotic resistance genes and plasmid replicons. The columns show name of the isolate; hatchery (Br – Brazil, Pa – Paraguay); farm (Villeta – V1-3; Nueva Italia NI1-2; Colonel Oviedo CO1-4; Paraguari – PA, Piribebuy – PI, Luque – L); phylogenetic group; sequence type; antibiotic resistance phenotype (resistance to: A – ampicillin, S – streptomycin, Su – sulphonamides, Te – tetracycline, Sxt – trimethoprim-sulfamethoxazole, C – chloramphenicol Kf, – cefalotin, Na – nalidixic acid, Caz – ceftazidime, Cn – gentamicin, Amc – amoxicillin-clavulanic acid, Cip – ciprofloxacin); purple squares indicates presence of antibiotic resistance genes; blue (F replicons) and turquoise squares indicates presence of plasmid replicons.

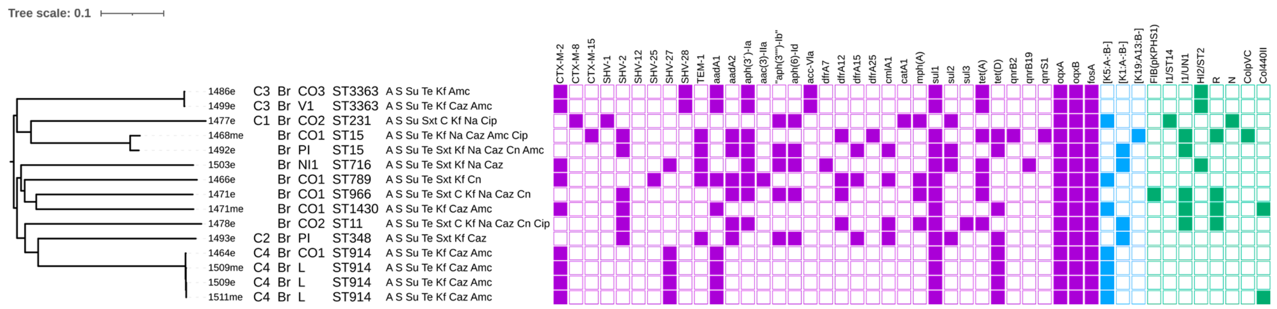

Isolate	Cluster	Breed	Farm	Phenotypic resistance	ESBL genes	Conjugation
1479e	C1	Br	CO2	A Su Te Sxt C Kf Na Cip	*bla*CTX-M-8	NP
1496e	C2	Br	PI	A S Su Te Sxt Kf Caz	*bla*SHV-2	NP
1482e	C3	Br	CO3	A S Su Te Kf	*bla*CTX-M-2	Yes
1465e	C4	Br	CO1	A S Su Te Kf Caz Amc	*bla*CTX-M-2, *bla*SHV-27	Yes
1469e	C4	Br	CO1	A S Su Te Kf Caz Amc	*bla*CTX-M-2, *bla*SHV-27	Yes
1470e	C4	Br	CO1	A S Su Te Kf Caz Amc	*bla*CTX-M-2, *bla*SHV-27	Yes
1511e	C4	Br	L	A S Su Te Kf Caz Amc	*bla*CTX-M-2, *bla*SHV-27	Yes

Figure S2. Phylogeny of selected *Klebsiella pneumoniae* isolates and table with data of non-sequenced *K. pneumoniae* isolates. The columns in phylogeny part show name of the isolate; cluster distinguished using pulsed-field gel electrophoresis (PFGE) the isolate belonged to (C1-C4; defined at 100% similarity level); hatchery (Br – Brazil); farm (Villeta – V1; Nueva Italia NI1; Colonel Oviedo CO1-3; Piribebuy – PI; Luque – L); sequence type, antibiotic resistance phenotype (resistance to: A – ampicillin, S – streptomycin, Su – sulphonamides, Te – tetracycline, Sxt – trimethoprim-sulfamethoxazole, C – chloramphenicol Kf, – cefalotin, Na – nalidixic acid, Caz – ceftazidime, Cn – gentamicin, Amc – amoxicillin-clavulanic acid, Cip – ciprofloxacin); purple squares indicates presence of antibiotic resistance genes; blue (F replicons) and turquoise squares indicates presence of plasmid replicons. The table part shows available data of non-sequenced *K. pneumoniae* isolates; the last column Conjugation indicates whether the conjugative transfer of extended-spectum beta-lactam (ESBL) encoding genes was successful (Yes) or not performed (NP).

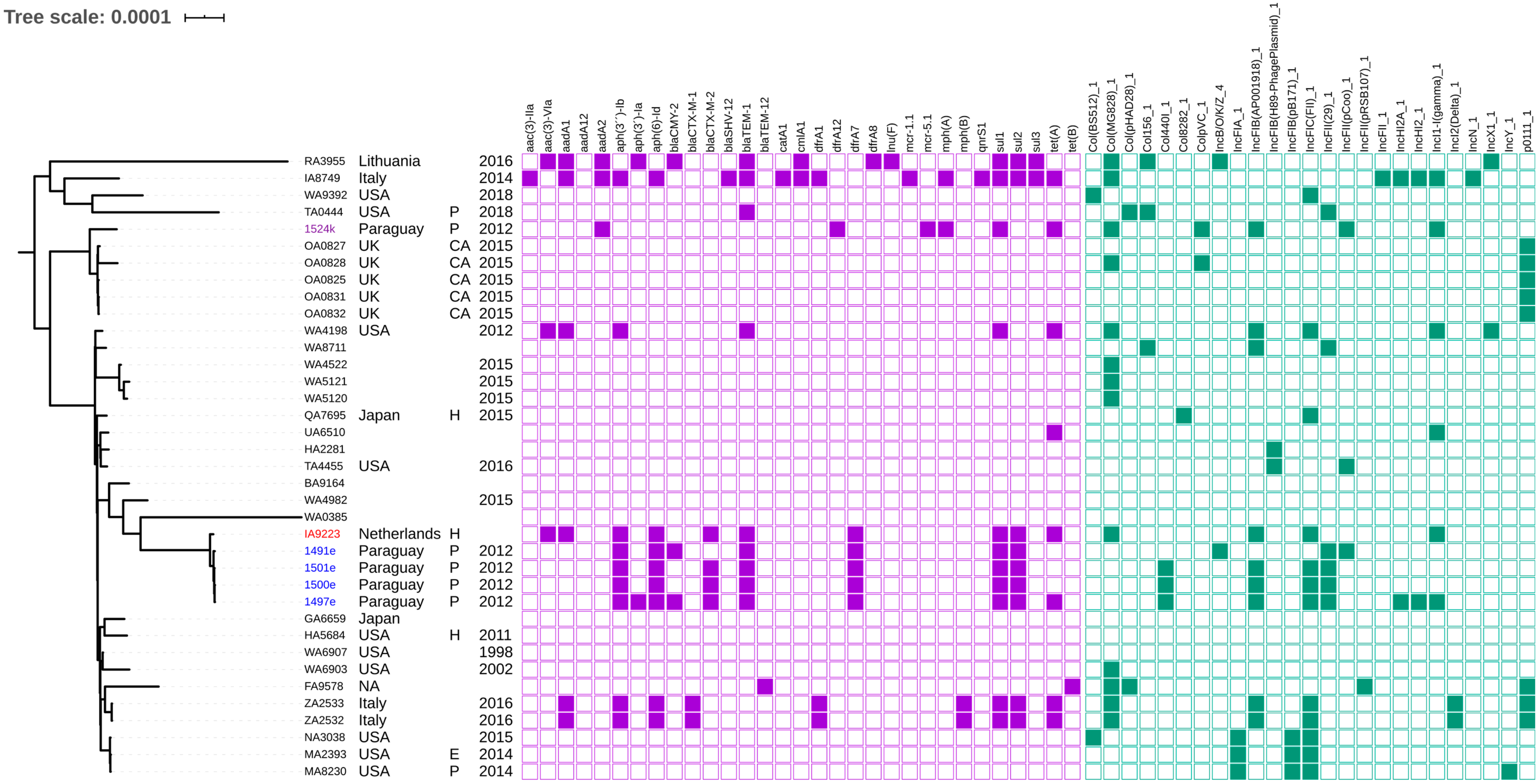

Figure S3. Phylogeny of *E. coli* sequence type (ST) 38 – comparison of our isolates with closely related ones recovered from EnteroBase. The sample names refer to original name of assemblies downloaded from EnteroBase. Our isolates from the Brazilian hatchery are highlighted in blue, an isolate from the Paraguayan hatchery is purple and a human strain SCP05-44 (ECS_EA9861AA, referred as IA9223 here) which is closely related (24 – 25 SNPs) to the Paraguayan strains is highlighted in red. The other columns reveal country of origin, source of origin (P – poultry; CA – companion animal, H – human, E – environment) and year of isolation. The purple squares represent presence of respective antibiotic resistance genes and the turquoise squares show presence of plasmid replicons.

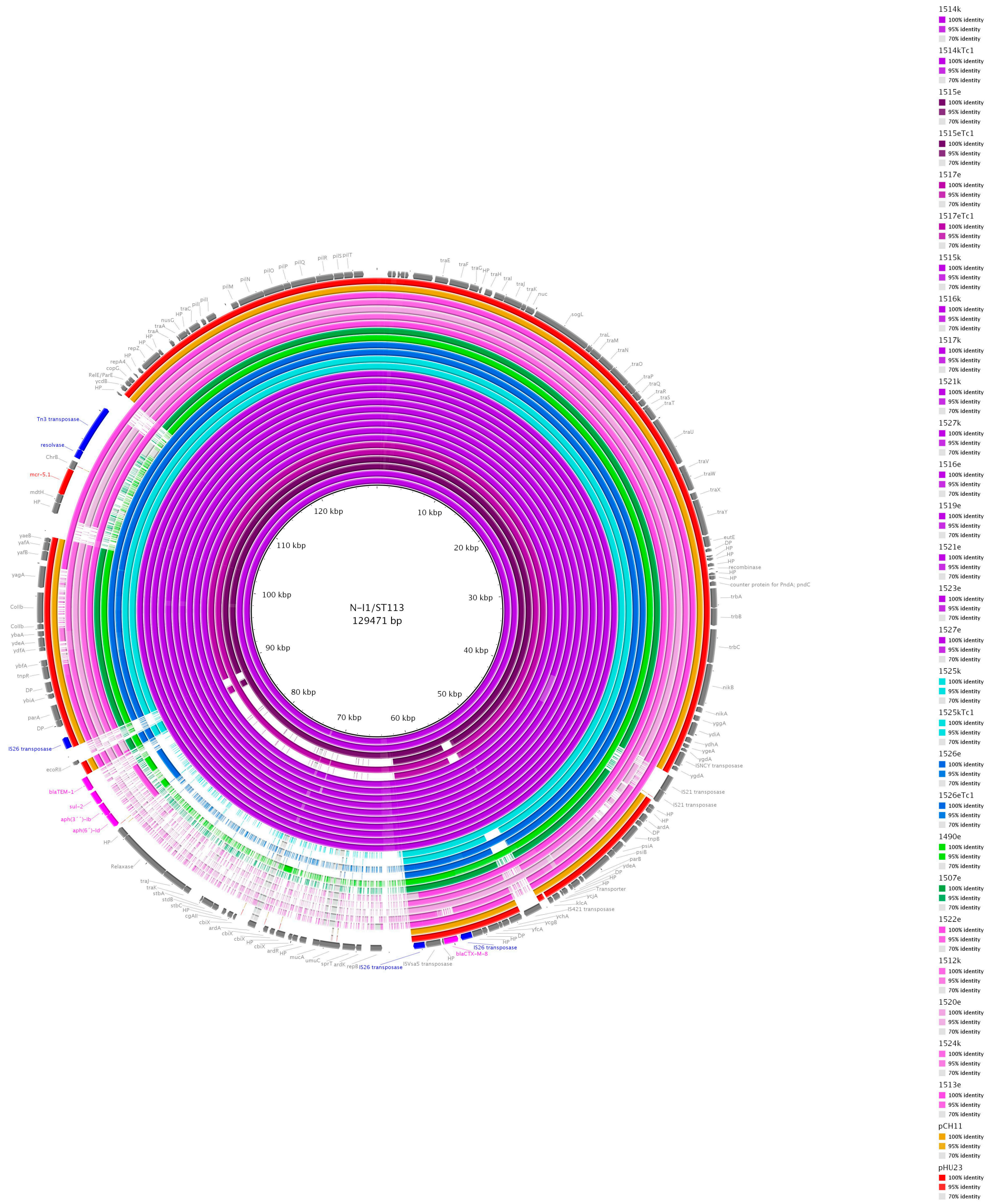

Figure S4. BRIG comparison of I1/ST113 plasmids. The reference plasmid 1514kTc1 was a fusion N-I1/ST113 (GenBank MW800641). The purpose of this figure is to compare all I1/ST113 plasmids we have detected and to reveal complication which may occur when whole-genome sequencing (WGS) data are used for BRIG plasmid alignment. For this purpose – five whole-genome sequences were aligned with the respective single-plasmid transconjugant plasmid DNA-sequence (marked as Tc1) which revealed that for *E. coli* ST457 isolates which all carried N replicon, using WGS data make incorrect impression that all the I1/ST113 plasmid are fusion ones. The incorrectness was revealed using S1-PFGE for selected transconjugants and polymerase-chain reaction testing of N replicon presence in all of *E. coli* ST457 transconjugants. The comparison also showed that transposon with *mcr-5* (red), was mapped from WGS data of isolates 1512k, 1520e and 1524k which carried it but not within the I1/ST113 plasmids.

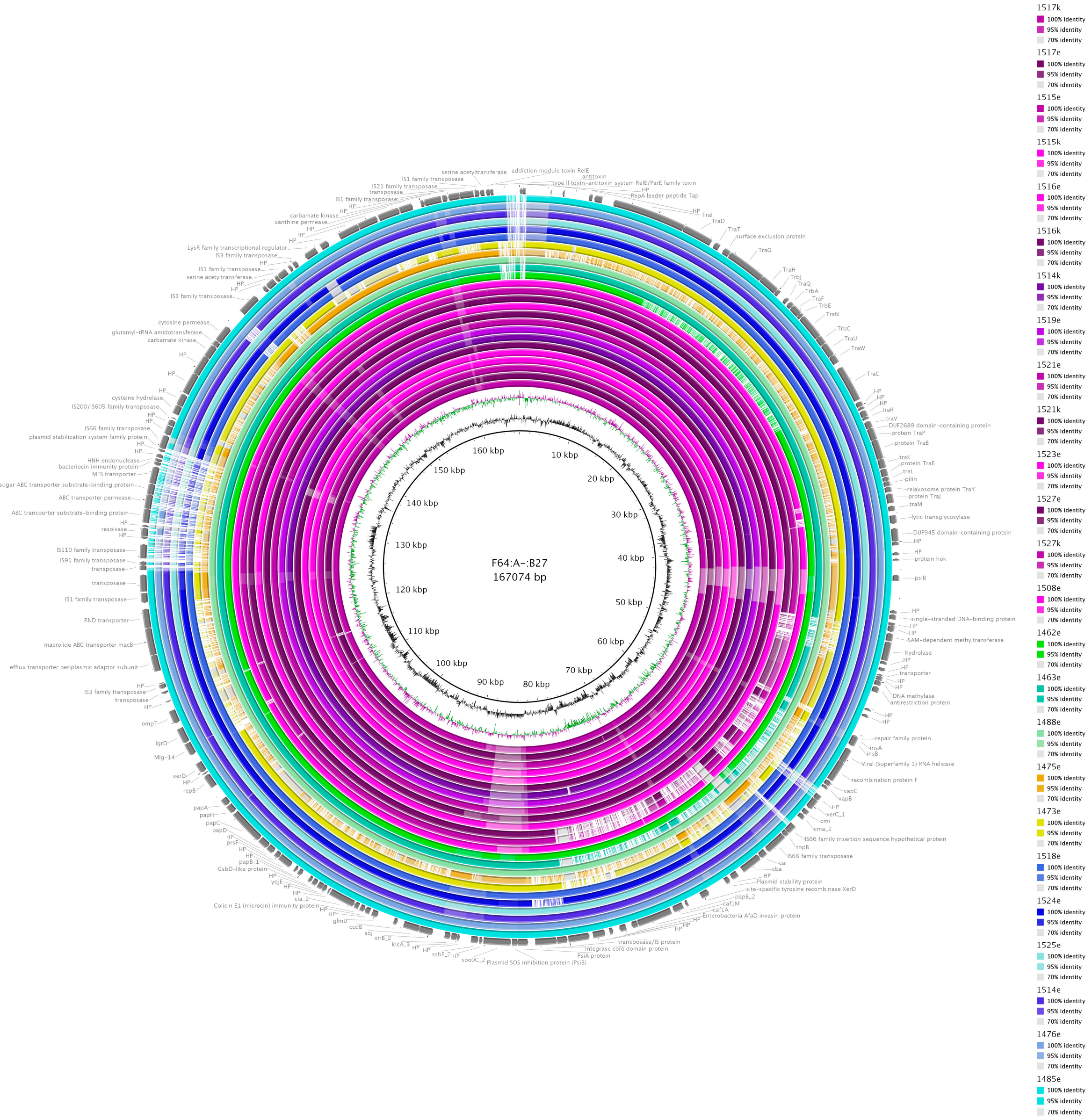

Figure S5. BRIG comparison of F64:A-:B27 plasmids. The reference plasmid F64:A-:B27 (GenBank MW800638) was obtained using Unicycler from PacBio and Illumina data of 1517k strain. Purple circles represents F64:A-:B27 plasmids found in *E. coli* ST457 isolates, green circles are from *E. coli* ST57, yellow from ST1158 and ST68 and blue from ST8061.

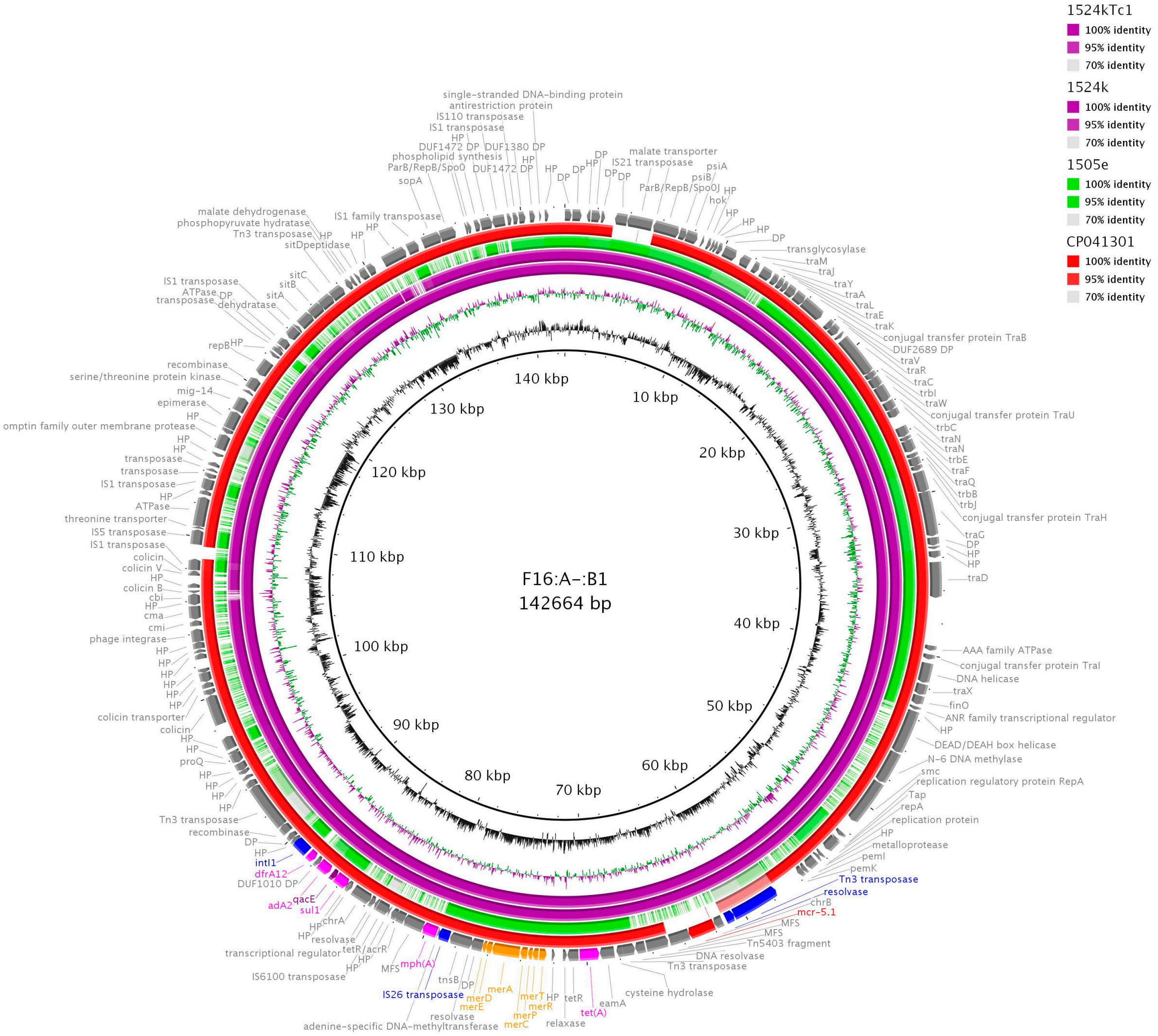

Figure S6. BRIG comparison of F16 replicon plasmids in this study with closely related F16 plasmid recovered from GenBank. The reference plasmid F16:A:B1 (GenBank MW800635) from 1524k carrying *mcr-5.1* was sequenced using PacBio, two purple circles represent this plasmid recovered from different type of data. The most inner circle (1524kTc1) uses data from Illumina sequencing of plasmid DNA from single-plasmid transconjugant, the second purple circle (1524k) uses Illumina WGS data from the wild-type. This attitude was used to see the limitation of WGS data from wild-type as for the other compared F16:A-:B- plasmid from isolate 1505e (green) only this type of data was available. Plasmid CP041301 (red) was found using BLASTn as closely related to the reference 1524k plasmid, it did not carry *mcr-5.1* (red), but the other resistance genes (fuchsia) was present as well as the mercury resistance operon (orange).

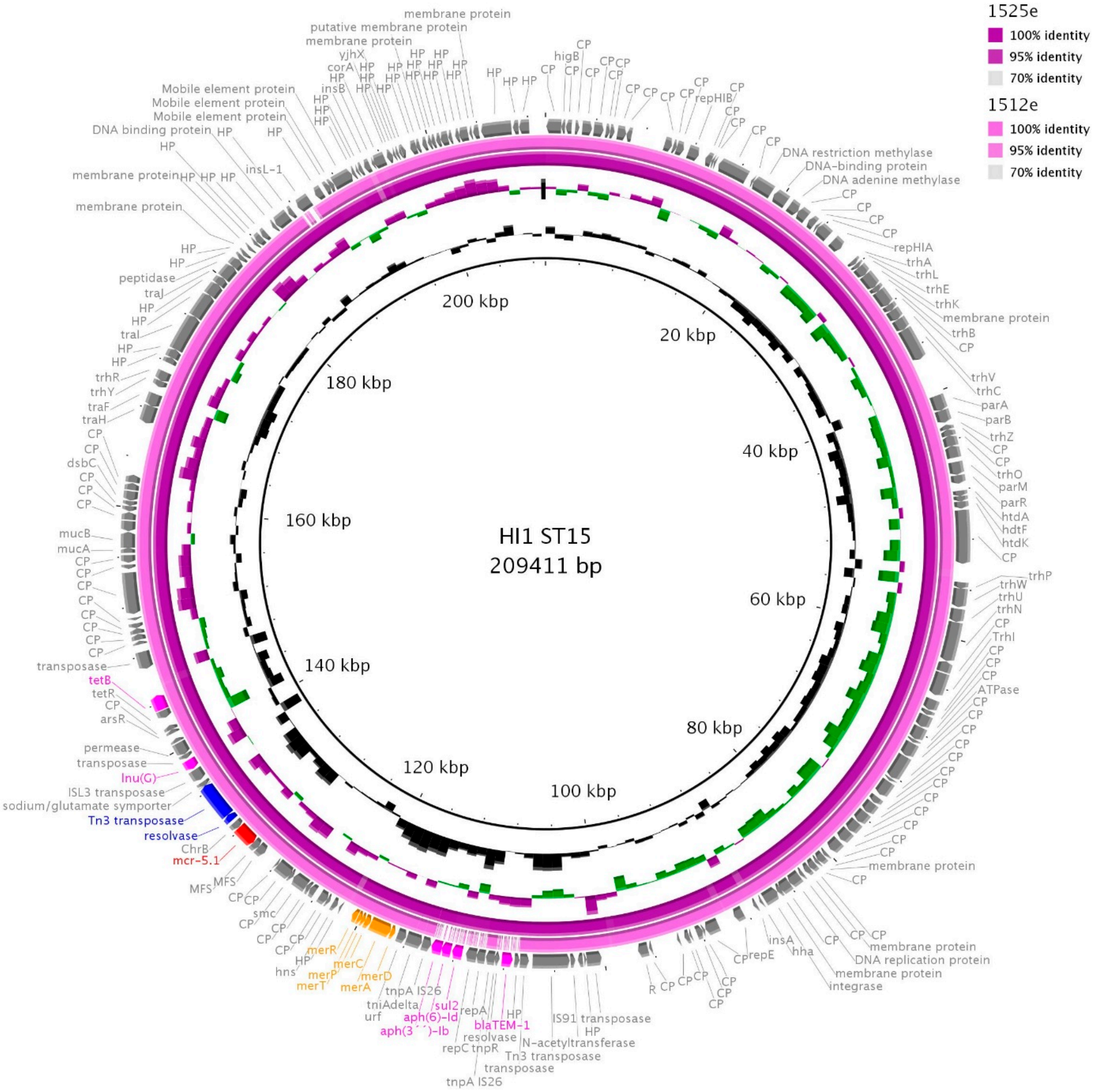

Figure S7. BRIG comparison of HI1/ST15 plasmids. Both HI1/ST15 plasmids were sequenced using PacBio and their comparison revealed they are very similar except the additional resistance region present in 1525e (GenBank MW800636) reference plasmid. The mobile elements of interest are highlighted in blue, *mcr-5* in red, other resistance genes in fuchsia and mercury resistance operon in orange.

Table S1: Comparison of 1517k (PacBio) and 1508e genomes in progressiveMauve.

Region	Position (1517k)	RAST Annotation
**A**	618531..619256	HTH-type transcriptional regulator gadW
	619552..620058	hypothetical protein
**B**	1334573..1335058	Antirestriction protein klcA
	1335150..1335968	UPF0380 proteins YafZ and homologs
	1336058..1336291	UPF0401 protein YkfF
**C**	1476145..1477404	N-acetylmuramoyl-L-alanine amidase (EC 3.5.1.28)
**D**	1834954..1835520	Fructose-1-phosphate phosphatase YqaB
	1835517..1835945	Inner membrane protein YqaA
	1836018..1837574	Glutamate--cysteine ligase
	1837724..1838239	S-ribosylhomocysteine lyase<br>Autoinducer-2 production protein LuxS
	1838303..1839841	Multidrug efflux system EmrAB-OMF,<br>inner-membrane proton/drug antiporter EmrB (MFS type)
	1839858..1841030	Multidrug efflux system EmrAB-OMF, membrane<br>fusion component EmrA
	1841007..1841129	FIG00638175: hypothetical protein
	1841157..1841687	Multidrug resistance regulator EmrR (MprA)
	1841778..1842113	Uncharacterized protein YgaH
	1842103..1842840	Inner membrane protein YgaZ
	1842964..1844148	Uncharacterized MFS-type transporter STM2812
	1844141..1844287	hypothetical protein
	1844340..1845332	Glycine betaine/L-proline transport<br>substrate-binding protein ProX
	1845390..1846454	Glycine betaine/L-proline transport systém<br>permease protein ProW
	1846447..1847649	Glycine betaine/L-proline transport ATP-binding<br>protein ProV
	1848005..1848964	Ribonucleotide reductase of class Ib (aerobic),<br>beta subunit (EC 1.17.4.1)"
	1848974..1851118	Ribonucleotide reductase of class Ib (aerobic),<br>alpha subunit (EC 1.17.4.1)
	1851091..1851501	Ribonucleotide reduction protein NrdI
	1851498..1851743	Glutaredoxin-like protein NrdH, required for<br>reduction of Ribonucleotide reductase class Ib
	1851952..1852383	hypothetical protein
	1852471..1853805	Transcriptional regulator, GntR family domain /<br>Aspartate aminotransferase
	1853862..1854191	ElaB protein
	1854343..1854687	Uncharacterized protein YgaC
	1854724..1855173	L-alanine exporter AlaE
	1855299..1855427	FIG00640544: hypothetical protein
	1855840..1856244	DNA-binding protein StpA
	1856291..1856815	Rhodanese-domain-containing inner membrane<br>protein YgaP

	1856825..1857124	Transcriptional regulator YgaV, ArsR family
	1857307..1857465	UPF0057 membrane protein YqaE
	1857549..1857998	Uncharacterized LysM domain protein YgaU
	1857999..1858661	CsiR, transcriptional repressor of CsiD
	1858682..1860082	gamma-aminobutyrate (GABA) permease
	1860319..1861599	Gamma-aminobutyrate:alpha-ketoglutarate aminotransferase
	1861613..1863061	Succinate-semialdehyde dehydrogenase [NAD(P)+]
	1863084..1864352	L-2-hydroxyglutarate oxidase
	1864372..1865349	Carbon starvation induced protein CsiD
	1865686..1867938	Alpha-amylase (EC 3.2.1.1) (1,4-alpha-D-glucan glucanohydrolase) (GLYCOGENASE)
**E**	1895379..1896707	Transposase InsG for insertion sequence element IS4
**F**	2016425..2017366	RecT protein
	2017363..2018184	RecT protein
**G**	2036167..2038680	Phage capsid and scaffold
	2038677..2040479	Phage protein
	2040485..2042959	Phage protein
	2043155..2043451	hypothetical protein
**H**	3708747..3709493	Colanic acid biosynthesis glycosyl transferase WcaE
